# Comparison of Cervical Cancer Screening Used between Individuals with Disabilities and Individuals without Disabilities

**DOI:** 10.3390/healthcare11101363

**Published:** 2023-05-09

**Authors:** Chia-Yu Chen, Pei-Tseng Kung, Li-Ting Chiu, Wen-Chen Tsai

**Affiliations:** 1 Graduate Institute of Public Health, China Medical University, Taichung 406040, Taiwan; u103050003@cmu.edu.tw; 2 Department of Healthcare Administration, Asia University, Taichung 41354, Taiwan; ptkung@asia.edu.tw; 3 Department of Medical Research, China Medical University Hospital, China Medical University, Taichung 404327, Taiwan; 4 Department of Health Services Administration, China Medical University, Taichung 406040, Taiwan; u9775851@cmu.edu.tw

**Keywords:** disability, pap smear test, women without disability, cervical cancer screening, women with disabilities

## Abstract

Objective: Cervical cancer is the fourth most prevalent cancer in women worldwide. It is vital to achieve a high cervical cancer screening rate among women. We compared the Pap smear test (PST) used between individuals with disabilities and those without disabilities in Taiwan. Methods: Individuals registered in the Taiwan Disability Registration File and the National Health Insurance Research Database (NHIRD) were screened for this nationally representative retrospective cohort study. Women aged 30 and above in 2016 and who were still alive in 2016 were matched in a 1:1 ratio via propensity score matching (PSM); 186,717 individuals with disabilities and 186,717 individuals without disabilities were included. Controlling for relevant variables, the odds of receiving PST were compared using conditional logistic regression analysis. Results: A lower percentage of individuals with disabilities (16.93%) received PST than those without disabilities (21.82%). The odds of individuals with disabilities receiving PST were 0.74 times that of individuals without disabilities (OR = 0.74, 95% CI = 0.73–0.76). Compared to individuals without disabilities, individuals with intellectual and developmental disabilities had the lower odds of receiving PST (OR = 0.38, 95% CI = 0.36–0.40), followed by individuals with dementia (OR = 0.40, 95% CI = 0.33–0.48) or multiple disabilities (OR = 0.52, 95% CI = 0.49–0.54). Conclusions: We highly recommend that healthcare practitioners recognize the unique needs of individuals with different types of disabilities, especially those with cognitive impairments.

## 1. Introduction

In the past decade, malignant tumors have consistently ranked as the leading cause of death, and cervical cancer is currently the fourth most frequent type of cancer among women worldwide [1]. It is also the first cancer that the World Health Organization (WHO) has committed to eliminating.

According to the Global Burden of Disease (GBD) report in 2020, there were 604,237 newly diagnosed cases of cervical cancer and 342,843 deaths [2]. The age-standardized incidence rate of cervical cancer was 13.3 per 100,000 people, with an average age of death at 59 years (ranging from 45 to 76 years) [3]. The incidence and mortality rates are higher in countries with a lower Human Development Index (HDI) [4]. The WHO has announced that reducing the prevalence of cervical cancer to lower than four in 100,000 would facilitate the elimination of cervical cancer and has proposed three strategies to eliminate cervical cancer, including increasing the Pap smear test (PST) rate [5]. Many studies have shown that regular PST at least once every three years has effectively reduced the incidence and mortality rates by 33–36% [6,7]. In Taiwan, the Ministry of Health and Welfare has provided free PST for women over 30 years old since 1995, and the screening rate reached 50.2% in 2018 [8]. The age-standardized incidence rate of cervical cancer in Taiwan is 7.9 per 100,000 women, with an average age at diagnosis of 58 years [9]. In Taiwan, the incidence rate has decreased by 70% compared to the past two decades [10]. However, many women still face inequalities in accessing healthcare services, such as those with disabilities. According to the WHO, there were over one billion people with disabilities worldwide in 2020, accounting for approximately 15% of the world’s population [11]. In the 2021 population statistics report in Taiwan, there were 1,203,756 people with disabilities, accounting for 5.15% of the population. Although this is lower than that in the global population, the highest proportion was among people over 65, indicating that the number of people with disabilities is increasing yearly due to population aging [12,13]. In Article 25 of the United Nations Convention on the Rights of Persons with Disabilities (CRPD) in 2008, it is emphasized that persons with disabilities should not be discriminated against and should have equal access to healthcare services [14]. However, studies have shown that compared to non-disabled individuals, persons with disabilities are less likely to receive invasive pelvic exams, especially those with functional limitations [15]. Furthermore, according to the latest survey reported by WHO, disabled people have up to six times more difficulty in accessing healthcare environment and equipment than non-disabled individuals [16].

Many studies have shown that numerous factors affect medical screening in women, including social and psychological barriers [17], cultural values [18], age, knowledge, screening guidelines [19], disability status [20,21], and severity of disabilities [22,23,24]. In addition, studies on screening for women with disabilities also revealed that marital status, economic status, degree of urbanization of the residence, and history of cancer diagnosis [25,26] are all factors influencing the screening rates. Previous studies on single disability categories showed that the PST rate among women with intellectual disabilities and mental disabilities was far lower (4.83% and 11.05%, respectively) than that among non-disabled individuals (28.8%) [27,28]. However, a regional survey study in Taiwan showed a more regular PST rate in women with physical disabilities than those without disabilities (74% vs. 64%) [29]. Due to this discrepancy and the fact that limited studies are comparing different disability categories, it is necessary to conduct comparative studies on the differences in PST for all disability categories. Therefore, this study compared the differences in receiving PST between women with and without disabilities in Taiwan.

## 2. Materials and Methods

### 2.1. Data Sources

This study extracted original data from the national disability registry, Ministry of Interior, Taiwan, and the National Health Insurance Research Database (NHIRD), the Ministry of Health and Welfare (MOHW), Taiwan. NHIRD has covered all residents since 1995 to ensure their right to medical insurance. The NHIRD system contains all medical care information for patients, with a coverage rate of 99.9% [30].

Both datasets are managed by the Ministry of Health and Welfare Statistics Center, and the personal privacy of the patients was protected, with all information encrypted and de-identified. The study was approved by the Institutional Review Board of Jen-Ai Hospital in Dali (IRB No: 105-14).

### 2.2. Research Participants

This study aimed to investigate the differences in receiving PST between women with and without disabilities. Since MOHW offers free annual PST for women aged 30 or older [31] in Taiwan, the study focused on whether women aged 30 or older received Pap smear screening in 2016.

According to the People with Disabilities Rights Protection Act in Taiwan, disabilities are classified into 16 categories, including moving functional limitation, internal organ function loss and related disabilities, chronic mental health conditions, hearing impairment, multiple disabilities, visual impairment, intellectual and developmental disability, dementia, vocal and speech impairment, motion and balance impairment, facial disfigurements, intractable epilepsy, rare diseases, congenital disorders, persistent vegetative state (PVS) and others (including autism, chromosomal abnormalities, and metabolic disorders). The study exclusion criteria included age under 30 years, women with a persistent vegetative state (PVS), and those who had suffered from any cancer before 2016. PVS individuals were excluded from this study because their long-term complete bedridden status did not meet the eligibility criteria.

**Figure 1:**
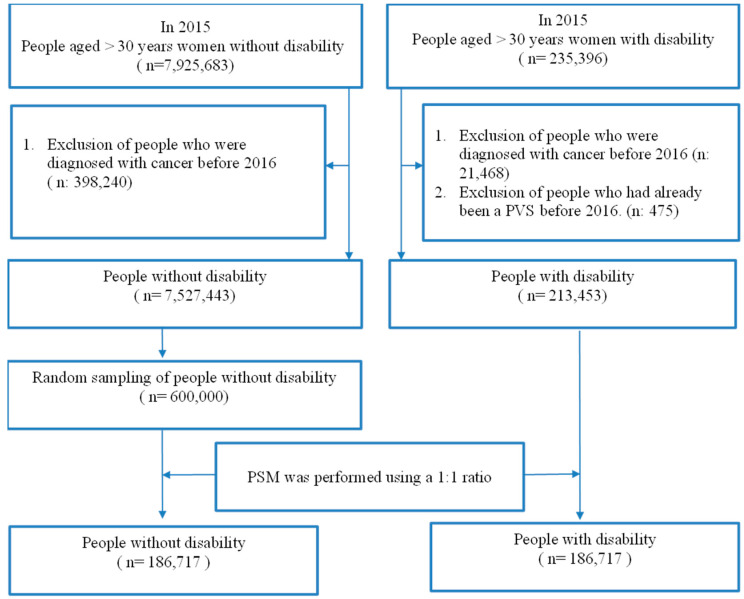
Flowchart of the selection of study participants.

A total of 235,396 individuals with disabilities and 7,925,683 individuals without disabilities participated in this study. The exclusion of 21,468 diagnosed with cancer before 2016 and 475 PVS women resulted in 213,453 disabled individuals. Among the individuals without disabilities, 398,240 diagnosed with cancer before 2016 were excluded, leaving 7,527,443 individuals. Since 7,527,443 women without disabilities comprised too large a dataset compared to women with disabilities (N = 213,453), we randomly selected 600,000 women from the 7,527,443 to be the control group. For this study, 600,000 individuals without disabilities were matched with disabled individuals by propensity score matching (PSM) at 1:1 in order to reduce between-group selection bias. As a result, 186,717 individuals without disabilities and 186,717 individuals with disabilities were finally included in this study, as shown in Figure 1.

### 2.3. Definition and Description of Variables

The variables in this study were as follows: independent variables (a) disability status: whether the participant was disabled (including those who meet the criteria for PST); (b) type of disabilities (15 categories): moving functional limitation, internal organ function loss and related disabilities, chronic mental health conditions, hearing impairment, multiple disabilities, visual impairment, intellectual and developmental disability, dementia, vocal and speech impairment, motion and balance impairment, facial disfigurements, intractable epilepsy, rare diseases, congenital disorders, and others (including autism, chromosomal abnormalities, and metabolic disorders); (c) severity of disability (four levels): mild, moderate, severe, and profound, which was categorized in the dataset. Dependent variables included whether the participant received PST. Control variables included: (a) essential characteristics (age): <45 years, 45–54 years, 55–64 years, 65–74 years, ≥75 years; (b) economic factors (monthly salary): five intervals: ≤NTD 17,280, NTD 17,281–22,800, NTD 22,801–28,800, NTD 28,801–36,300, and ≥NTD 36,301; (c) environmental factors: level of urbanization of the participant’s residence, divided into seven levels, with level 1 being the highest and level 7 being the lowest, which was conducted by Liu et al. [32]; (d) health status: the Charlson Comorbidity Index (CCI) was used to measure the severity of comorbidities by converting ICD-9-CM diagnosis codes into weighted scores, with scores of 0, 1, 2, and ≥3 indicating increasing severity of comorbidities [33]; (e) preventive healthcare and utilization. Taiwan did not provide the HPV vaccine in 2016, Taiwan’s national health insurance provides free biannual teeth cleaning. In addition, there is no age limit; therefore, this study took into consideration whether or not the participant received preventive teeth cleaning in 2016 as an indicator of their preventive healthcare behavior [34]. In addition, according to the database of National Health Insurance of the Taiwan Ministry of Health and Welfare, the procedure codes for identification of PST are No: 31, 35, IC 31, IC 35, and IC 37 [34].

### 2.4. Statistical Analysis

This study is a retrospective cohort study, and all statistical analyses were performed using SAS 9.4 (SAS Institute Inc., Cary, NC, USA)

Following the study’s objectives, the study first used PSM at a 1:1 ratio to match participants with and without disabilities (observation group and control group, respectively) based on variables including age, monthly income, urbanization level of the residence, and Charlson Comorbidity Index (CCI) to reduce selection bias between the two groups. Then, the chi-square test (χ^2^) was used to compare the utilization rate of PST between groups, *p* < 0.05 was considered significant, and further conditional logistic regression analysis was conducted with the inclusion of five control variables to compare the probability of PST between participants with and without disabilities and related factors. Finally, this study performed a stratified analysis for the severity of the disability.

## 3. Results

This study aimed to investigate the difference in the utilization rate of PST between participants with and without disabilities. This study employed a rigorous research design by conducting 1:1 PSM matching between the two groups, with 186,717 participants in each group, for a total of 373,434 participants included in the study (Figure 1). The test results showed no significant differences in the distribution of four variables, including age, monthly salary, urbanization level of the residence, and CCI, between the two groups (*p* > 0.05; Table 1).

Table 2 comparison of the use of PST by disabled and non-disabled individuals after matching. After testing, the proportion of disabled individuals who received PST (16.93%) was lower than that of non-disabled individuals (21.82%) (*p* < 0.05). In age analysis, the overall rate of PST among disabled individuals was lower than that of non-disabled individuals, especially those aged 75 years or older. Regarding economic income comparison, the proportion of disabled individuals who received PST was lower than that of non-disabled individuals, with the lowest proportion observed among those with an income of ≤NT$17,280. Regardless of the degree of urbanization in the living area, the proportion of disabled individuals who received PST was lower than that of non-disabled individuals. An analysis of the severity of comorbidities found that the proportion of disabled individuals who received PST was lower than that of non-disabled individuals and that the proportion of those who received screening decreased with increasing comorbidity severity (CCI). From the free preventive dental cleanings under the National Health Insurance System, we found that people with disabilities have lower rates of PST use than non-disabled people, regardless of whether this preventive health care behavior was available.

This study analyzed the proportion of PST used among various types of disabled individuals (Table 3). The results showed that facial disfigurements had the highest proportion of individuals who received PST (29.47%), followed by intractable epilepsy (28.08%). Dementia had the lowest proportion of individuals who received PST among all disabilities (4.30%). Among the severity rankings of disabilities, mild disability (20.41%) had the highest proportion of individuals who received PST.

In Table 4, after controlling for relevant variables, the results of conditional logistic regression analysis showed that the odds of receiving PST by individuals with disabilities were 0.74 times lower than those without disabilities (aOR = 0.74, 95% CI = 0.73–0.76, *p* < 0.05) (Model A). Further exploration of different types of disabilities among disabled individuals revealed that those with an intellectual and developmental disability had the lowest rates of PST utilization, which was 0.38 times that of non-disabled individuals (aOR = 0.38, 95% CI = 0.36–0.40). Dementia had the second lowest rate of PST utilization, which was 0.40 times that of non-disabled individuals (aOR = 0.40, 95% CI = 0.33–0.48), and multiple disabilities, which was 0.52 times that of non-disabled individuals (aOR = 0.52, 95% CI = 0.49–0.54). However, facial disfigurements and intractable epilepsy were found to have higher rates of PST utilization compared to non-disabled individuals, with odds ratios of 1.18 (95% CI = 0.99–1.40) and 1.06 (95% CI = 0.88–1.27), respectively (Model B), which were not statistically significant (*p* > 0.05). In addition, when comparing the severity of disabilities with non-disabled individuals, the results showed that the utilization rate of PST by individuals with disabilities decreased with increasing severity of disabilities. For example, the utilization rate for individuals with mild disabilities was 0.94 times that of non-disabled individuals (aOR = 0.92, CI = 0.90–0.96), In comparison, the utilization rate for individuals with profound disabilities was 0.48 times that of non-disabled individuals (aOR = 0.48, CI = 0.46–0.51) (Model C).

We performed the stratified analysis for the severity of disability. The results showed that women with mild disabilities with different types of impairments had higher odds of using cervical cancer screening compared to those without disabilities. Since some sample sizes were too small, we combined several types of disability groups into one group called “others”. The results are shown in the Supplementary Table S1.

## 4. Discussion

In many previous studies on PST use, the focus was primarily on comparing individuals with a single physical or mental disability with those without disabilities or comparing individuals with specific diseases to the general female population. This study included 15 categories of physical and mental disabilities (excluding PVS) and the severity level of the disability, making the results more comprehensive and representative.

To ensure the rigor of the research, this study used propensity score matching (PSM) in advance to reduce selection bias between the two groups. The study found that even with a national coverage rate of more than 99.9% and a large-scale promotion of PST policy under Taiwan’s National Health Insurance, only 16.93% of individuals with disabilities received regular PST. The study also found significant differences among disability types. For example, facial disfigurements had the highest proportion of individuals who received PST (29.47%), followed by intractable epilepsy (28.08%). Conversely, dementia had the lowest proportion of individuals who received PST among all disabilities (4.30%). Overall, the odds of individuals with disabilities receiving PST were 0.7 times that of individuals without disabilities (aOR = 0.74), consistent with the results of most relevant studies in other countries [35,36,37,38].

Another Taiwanese regional survey study in a Taiwan study [29] showed the opposite results to this study: the proportion of disabled individuals receiving PST was higher than that of non-disabled individuals (74% vs. 64% for those aged ≥30). Further analysis revealed that this previous study only targeted disabled individuals aged 15 or above in a specific city in Taiwan. The results may not be representative due to the email survey method, which may have affected the accuracy of responses and introduced bias [29]. In addition, a ten-year study in the U.K. found that over time, the difference in PST rates between disabled and non-disabled individuals decreased but ultimately remained lower for disabled individuals [39], which is consistent with the report by the World Health Organization (WHO) that disabled individuals experience more inequalities in healthcare access and services. Still, these issues can improve through various health policies and implementation plans [40].

This study further analyzed different categories of disabilities and found that inequality exists not only between people with and without disabilities but also between different types of disabilities. Compared to individuals without disabilities, intellectual and developmental disability (aOR = 0.38), dementia (aOR = 0.40), and multiple disabilities (aOR = 0.52), they had the lowest odds of PST. In contrast, facial disfigurements (aOR = 1.18) and intractable epilepsy (aOR = 1.06) had the highest odds of PST among all disability types and were similar to those of individuals without disabilities. This empirical study found that the health inequality among people with disabilities is consistent with the World Health Organization’s statement that the health of people with disabilities worldwide is significantly lower than that of those without disabilities [41].

The US NHIS survey on PST found that the probability of undergoing PST among people with mobility limitations is 0.7 times that of individuals without disabilities. When comparing different types of disabilities based on mobility limitations and sensory, social, mental, or cognitive impairments, the probability of undergoing PST was 0.5 times lower than that of individuals without disabilities. Furthermore, compared to individuals with mobility limitations, other people with disabilities were even less likely to undergo PST [35].

In addition, other studies have shown that regardless of having health insurance, individuals with disabilities have more health problems that require care compared to those without disabilities [42]. However, in reality, individuals with disabilities are less likely to receive necessary healthcare services compared to the general population, especially those with lower limb and cognitive impairments, who are even less likely to access healthcare services [43]. For example, a group of Canadian physicians stated in an interview study that healthcare access is equally important for individuals with and without disabilities. However, due to the diverse range of disabilities and complex needs of some individuals, healthcare providers may spend too much time and resources on these patients. As a result, the medical staff chooses to give up providing healthcare services to the disabled eventually. For example, doctors may feel that individuals with cognitive impairments cannot understand the rationale behind screening or tolerate invasive pelvic exams, so it is not recommended to perform PST on patients with dementia because pelvic exams can cause confusion and fear [44]. Scholars Bussières et al. (2015), in order to better understand possible determinants of women undergoing Pap smears conducted a large-scale survey of adults living with disabilities, which showed that people with more significant functional and cognitive limitations have a lower screening rate for Pap smears, and that disability severity is a barrier to cancer screening even if they live in a nursing home or have access to a well-resourced health system. The coverage of the scans also varies [45]. In this study, however, we performed the stratified analysis for the severity of disability. The results showed that women with mild disabilities with different types of impairments had higher odds of using cervical cancer screening compared to those without disabilities. Unfortunately, this also means that it may be more difficult for people with severe disabilities to undergo Pap smear screening.

According to the World Health Organization and healthcare providers, everyone should have access to the same preventative healthcare services, regardless of whether or not they have disabilities. To protect the healthcare rights and interests of individuals with disabilities, especially the increasing number of dementia patients, Taiwan is taking measures in the areas of law, policy, and the environment. They are also setting goals for the education and training of service providers to strengthen the training of healthcare professionals and improve communication skills for dementia care services [46] to eliminate obstacles as much as possible and ensure that the needs of individuals with disabilities can be understood and accepted. In that case, it will be helpful to reduce the health inequalities caused by disparities [47].

In addition, it is worth mentioning that some studies have shown that the older the individuals with disabilities, the lower the rate of PST [35], as evidenced by the results of this study, which showed that the lowest rates of PST were among those aged 65–74 years (16.55%) and >75 years (3.82%). In 2020, the American Cancer Society updated its guidelines on PST, recommending that women over 65 with a completely normal screening history in the past ten years should stop all PST [48]. The National Cancer Institute (NCI) also supports this recommendation. The U.S. Preventive Services Task Force (USPSTF) further recommends that women with regular screening results for over 20 years do not need to resume PST, even if they have a new sexual partner. These updated guidelines can help create more targeted healthcare screening policies for countries with an aging population and reduce unnecessary medical waste. However, the average cervical cancer incidence rate in all countries is 59 years old, similar to the rate of 58 years old in Taiwan. Therefore, increasing the screening rate for the 45–54 and 55–64 age groups is suggested to achieve the goal of actively eliminating cervical cancer, as set by WHO.

Since the data and information in this study come from secondary sources, research participants’ self-awareness, social psychology, cultural values, knowledge, and attitude information were unavailable. However, research shows that having a positive attitude and wisdom is more likely to lead to acceptance of PST. The results of this study show that there may be differences in PST rates between individuals with and without disabilities, which may be related to differences in knowledge, attitudes, cultural values, or social psychology. Therefore, future studies on PST may consider including these variables.

This study validates the results for different disability categories, which will help us understand more precisely the barriers that may need to be overcome or the need for enhanced care among women with other disabilities. However, there are still some limitations in the actual situation. According to the contemporary disability framework described in Jette’s paper [49], not all impairments or functional limitations result in disability, and disability may or may not be due to the interaction of an individual’s physical or mental limitations with social and biological factors in the individual’s environment. Patients with similar underlying pathologies, impairments, and functional limitations may exhibit different disability characteristics. Additionally, different types of health conditions may result in similar patterns of disability. These essential concepts are worthy of further reflection when we evaluate disabled patients.

### Strengths and Limitations

This research has several strengths. First, Taiwan’s health insurance coverage rate exceeds 99.9%, and the National Health Insurance Administration has a complete database that can provide reasonably accurate statistical data analysis. Second, this study used propensity score matching (PSM) to limit the selection bias and make a more reasonable comparison between the two groups. Third, this study compared women without disabilities with those with different categories of physical and mental disabilities, comparing the four severity levels. Also, this study has some limitations. First, it adopted a retrospective design and used secondary data, which cannot capture the study participants’ knowledge, cognitive abilities, and attitudes. Second, other countries’ health insurance systems differ from Taiwan’s, so the results may not be generalizable to other countries. Finally, although we compared disabilities by category, this classification comprised various impairments, health conditions, and one moving functional limitation. According to the contemporary disablement frameworks described in Jette’s paper [49], the disability classification applied in this study still has some limitations. For instance, patients with a similar underlying pathology, impairments, and functional limitations could present very different disability profiles. On the other hand, similar patterns of disability may be caused by various health conditions. All of these factors may influence the healthcare behavior of women undergoing Pap smear screening.

## 5. Conclusions

This study showed that the rate of PST among women with disabilities in Taiwan is lower than that of women without disabilities. Intellectual and developmental disabilities, dementia, and multiple disabilities denoted the weakest rates of PST among all disabilities. In addition, factors such as advanced age, low economic income, and the severity of the disability are associated with lower PST rates. Based on the results, healthcare providers should recognize that disabled individuals have special needs and barriers, especially those with intellectual and cognitive impairments. Furthermore, our results can help to guide future public health policies in this area.

## Figures and Tables

**Table 1:** Distribution after Matching between People with and without Disabilities.

	N	%	Without Disability		With Disability		*p*-Value ^a^
			N	%	N	%	
Total	373,434	100.00	186,717	50.00	186,717	50.00	
Age (years)							0.890
<45	67,838	18.17	33,828	18.12	34,010	18.21	
45–54	72,169	19.33	36,029	19.30	36,140	19.36	
55–64	86,861	23.26	43,512	23.30	43,349	23.22	
65–74	65,003	17.41	32,510	17.41	32,493	17.40	
≥75	81,563	21.84	40,838	21.87	40,725	21.81	
Monthly salary (NT$ ^b^)							0.985
≤17,280	113,414	30.37	56,626	30.33	56,788	30.41	
17,281–22,800	150,395	40.27	75,267	40.31	75,128	40.24	
22,801–28,800	23,388	6.26	11,693	6.26	11,695	6.26	
28,801–36,300	26,655	7.14	13,327	7.14	13,328	7.14	
≥36,301	59,582	15.96	29,804	15.96	29,778	15.95	
Urbanization level of residence area							0.988
Level 1	81,276	21.76	40,586	21.74	40,690	21.79	
Level 2	112,073	30.01	56,014	30.00	56,059	30.02	
Level 3	65,441	17.52	32,822	17.58	32,619	17.47	
Level 4	63,870	17.10	31,920	17.10	31,950	17.11	
Level 5	11,232	3.01	5625	3.01	5607	3.00	
Level 6	20,207	5.41	10,106	5.41	10,101	5.41	
Level 7	19,335	5.18	9644	5.17	9691	5.19	
CCI ^c^							0.887
0	199,592	53.45	99,839	53.47	99,753	53.42	
1	82,480	22.09	41,263	22.10	41,217	22.07	
2	50,784	13.60	25,402	13.60	25,382	13.59	
≥3	40,578	10.87	20,213	10.83	20,365	10.91	
Dental calculus cleaning							<0.001
No	565,562	69.52	395,964	65.99	169,598	79.44	
Yes	247,921	30.48	204,036	34.01	43,885	20.56	

a. Chi-square test; b. NT$: New Taiwan dollar; c. CCI: Charlson comorbidity ind.

**Table 2:** Comparison of the Use of Pap smear for PST between People with and without Disability after Matching.

	N	%	Without Disability (N = 186,717)				With Disability (N = 186,717)				*p*-Value
			No Pap	Pap ^a^			No Pap	Pap			
			N	%	N	%	N	%	N	%	
Total	373,434	100.00	145,969	78.18	40,748	21.82	155,099	83.07	31,618	16.93	<0.001
Age (years)											
<45	67,838	18.17	24,618	72.77	9210	27.23	28,488	83.76	5522	16.24	<0.001
45–54	72,169	19.33	25,395	70.48	10,634	29.52	27,427	75.89	8713	24.11	<0.001
55–64	86,861	23.26	31,508	72.41	12,004	27.59	32,899	75.89	10,450	24.11	<0.001
65–74	65,003	17.41	25,759	79.23	6751	20.77	27,114	83.45	5379	16.55	<0.001
≥75	81,563	21.84	38,689	94.74	2149	5.26	39,171	96.18	1554	3.82	<0.001
Monthly salary (NT$ ^b^)											
≤17,280	113,414	30.37	45,984	81.21	10,642	18.79	49,409	87.01	7379	12.99	<0.001
17,281–22,800	150,395	40.27	58,533	77.77	16,734	22.23	62,159	82.74	12,969	17.26	<0.001
22,801–28,800	23,388	6.26	8729	74.65	2964	25.35	9135	78.11	2560	21.89	<0.001
28,801–36,300	26,655	7.14	9949	74.65	3378	25.35	10,503	78.80	2825	21.20	<0.001
≥36,301	59,582	15.96	22,774	76.41	7030	23.59	23,893	80.24	5885	19.76	<0.001
Urbanization level of residence area											
Level 1	81,276	21.76	31,528	77.68	9058	22.32	33,624	82.63	7066	17.37	<0.001
Level 2	112,073	30.01	43,467	77.60	12,547	22.40	46,285	82.56	9774	17.44	<0.001
Level 3	65,441	17.52	25,835	78.71	6987	21.29	27,469	84.21	5150	15.79	<0.001
Level 4	63,870	17.10	25,351	79.42	6569	20.58	26,842	84.01	5108	15.99	<0.001
Level 5	11,232	3.01	4434	78.83	1191	21.17	4660	83.11	947	16.89	<0.001
Level 6	20,207	5.41	7873	77.90	2233	22.10	8224	81.42	1877	18.58	<0.001
Level 7	19,335	5.18	7481	77.57	2163	22.43	7995	82.50	1696	17.50	<0.001
CCI ^c^											
0	199,592	53.45	77,154	77.28	22,685	22.72	82,263	82.47	17,490	17.53	<0.001
1	82,480	22.09	31,788	77.04	9475	22.96	33,669	81.69	7548	18.31	<0.001
2	50,784	13.60	20,057	78.96	5345	21.04	21,283	83.85	4100	16.15	<0.001
≥3	40,578	10.87	16,970	83.96	3243	16.04	17,885	87.82	2480	12.18	<0.001
Dental calculus cleaning											
No	279,706	74.90	108,338	81.38	24,794	18.62	125,483	85.61	21,091	14.39	<0.001
Yes	93,728	25.10	37,631	70.23	15,954	29.77	29,616	73.78	10,527	26.22	<0.001

a. Pap smear Test; b. New Taiwan dollar; c. Charlson comorbidity index.

**Table 3:** The use of Pap smear for screening cervical cancer in people with and without disability.

	N	%	No Pap		Pap ^a^		*p*-Value
			N	%	N	%	
Total	373,434	100.00	301,068	80.62	72,366	19.38	
Without disability	186,717	50.00	145,969	78.15	40,748	21.82	<0.001
With disability	186.717	50.00	155,099	83.07	31,618	16.93	
Disability type							
Without disability	186,717	50.00	145,969	78.18	40,748	21.82	<0.001
Moving functional limitation	79,320	21.24	65,238	82.25	14,082	17.75	
Internal organ function loss and related disabilities	14,940	4.00	12,242	81.94	2698	18.06	
Chronic mental health conditions	25,137	6.73	19,497	77.56	5640	22.44	
Hearing impairment	19,730	5.28	16,463	83.44	3267	16.56	
Multiple disabilities	13,160	3.52	11,559	87.83	1601	12.17	
Visual impairment	11,296	3.02	9663	85.54	1633	14.46	
Intellectual and developmental disability	16,568	4.44	14,801	89.33	1767	10.67	
Dementia	2723	0.73	2606	95.70	117	4.30	
Vocal and speech impairment	1803	0.48	1454	80.64	394	19.36	
Motion and balance impairment	363	0.10	318	87.60	45	12.40	
Facial disfigurements	621	0.17	438	70.53	183	29.47	
Intractable epilepsy	577	0.15	415	71.92	162	28.08	
Rare diseases	128	0.03	99	77.34	29	22.66	
Congenital disorders	120	0.03	101	84.17	19	15.83	
Others ^b^	231	0.06	205	88.74	26	11.26	
Severity of disability							
Without disability	186,717	50.00	145,969	78.18	40,748	21.82	<0.001
Mild	76,488	20.48	60,877	79.59	15,611	20.41	
Moderate	64,960	17.40	54,509	83.91	10,451	16.09	
Severe	28,303	7.58	24,948	88.15	3355	11.85	
Profound	16,966	4.54	14,765	87.03	2201	12.97	

a. Pap smear Test; b. chromosomal abnormalities, inborn errors of metabolism, and autism.

**Table 4:** Conditional Logistic Regression Results: the use of Pap smear for cervical cancer screening in people with and without a disability.

	Model A ^a^				Model B ^a^				Model C ^a^			
	aOR	95%CI		*p*-Value	aOR	95% CI		*p*-Value	aOR	95% CI		*p*-Value
Disability												
Without (ref)	1.00	-	-	-								
With	0.74	0.73	0.76	<0.001								
Disability type												
Without disability (ref)					1.00							
Moving functional limitation					0.79	0.77	0.80	<0.001				
Internal organ function loss and related disabilities					0.74	0.71	0.78	<0.001				
Hearing impairment					0.95	0.91	0.99	<0.001				
Multiple disabilities					0.52	0.49	0.54	<0.001				
Chronic mental health conditions					0.89	0.86	0.92	<0.001				
Visual impairment					0.78	0.74	0.83	<0.001				
Intellectual and developmental disability					0.38	0.36	0.40	<0.001				
Dementia					0.40	0.33	0.48	<0.001				
Vocal and speech impairment					0.76	0.67	0.85	<0.001				
Motion and balance impairment					0.59	0.43	0.81	0.001				
Facial disfigurements					1.18	0.99	1.40	0.069				
Intractable epilepsy					1.06	0.88	1.27	0.572				
Rare diseases					0.82	0.54	1.25	0.363				
Congenital disorders					0.52	0.32	0.86	0.011				
Other ^b^					0.34	0.23	0.52	<0.001				
Severity of disability												
Without disability (ref)									1.00			
Mild									0.94	0.92	0.96	<0.001
Moderate									0.70	0.68	0.72	<0.001
Severe									0.52	0.50	0.54	<0.001
Profound									0.48	0.46	0.51	<0.001
Age (years)												
<45	1.00	-	-	-								
45–54	1.25	1.22	1.28	<0.001	1.18	1.15	1.21	<0.001	1.24	1.21	1.27	<0.001
55–64	1.15	1.12	1.18	<0.001	1.07	1.05	1.10	<0.001	1.13	1.10	1.15	<0.001
65–74	0.75	0.73	0.77	<0.001	0.69	0.67	0.71	<0.001	0.72	0.77	0.74	<0.001
≥75	0.16	0.15	0.16	<0.001	0.15	0.14	0.15	<0.001	0.15	0.14	0.16	<0.001
Monthly salary (NT$ ^b^)												
≤17,280 (ref) household	1.00											
17,281–22,800	1.42	1.39	1.45	<0.001	1.38	1.35	1.41	<0.001	1.37	1.34	1.40	<0.001
22,801–28,800	1.45	1.40	1.50	<0.001	1.40	1.35	1.45	<0.001	1.39	1.34	1.44	<0.001
28,801–36,300	1.47	1.42	1.52	<0.001	1.42	1.37	1.47	<0.001	1.42	1.37	1.47	<0.001
≥36,301	1.44	1.40	1.48	<0.001	1.39	1.36	1.43	<0.001	1.39	1.36	1.43	<0.001
Urbanization level of residence area												
Level 1(ref)	1.00											
Level 2	1.02	1.00	1.05	0.044	1.03	1.00	1.05	0.021	1.02	1.00	1.05	0.051
Level 3	1.00	0.98	1.03	0.797	1.01	0.98	1.04	0.413	1.01	0.98	1.03	0.659
Level 4	1.06	1.03	1.09	<0.001	1.07	1.04	1.10	<0.001	1.06	1.03	1.09	<0.001
Level 5	1.29	1.22	1.36	<0.001	1.31	1.24	1.38	<0.001	1.28	1.22	1.35	<0.001
Level 6	1.38	1.33	1.44	<0.001	1.40	1.34	1.46	<0.001	1.38	1.32	1.43	<0.001
Level 7	1.27	1.21	1.32	<0.001	1.28	1.23	1.34	<0.001	1.26	1.21	1.31	<0.001
CCI ^c^												
0	1.00											
1	1.30	1.27	1.33	<0.001	1.30	1.27	1.33	<0.001	1.30	1.27	1.33	<0.001
2	1.24	1.21	1.28	<0.001	1.25	1.21	1.28	<0.001	1.30	1.26	1.33	<0.001
≥3	1.08	1.05	1.12	<0.001	1.09	1.06	1.13	<0.001	1.15	1.11	1.19	<0.001
Dental calculus cleaning												
No	1.00											
Yes	1.67	1.64	1.70	<0.001	1.65	1.62	1.68	<0.001	1.65	1.62	1.68	<0.001

a. Three Model A, Model B, and Model C controlled for five control variables, including age, monthly salary, urbanization level of the residential area, comorbidity severity, and dental calculus cleaning; b. New Taiwan dollars; c. Charlson comorbidity index.

## Data Availability

Taiwan’s Ministry of Health and Welfare (MOHW) provides data through its Health and Welfare Data Science Center (https://www.mohw.gov.tw/mp-2.html (accessed on 1 April 2023). The MOHW manages a database that is available to all researchers. In accordance with Taiwan’s Personal Information Protection Act, public access to the database is not permitted. Therefore, the authors were not able to make this data set publicly available.

## Supplementary Materials

The following supporting information can be downloaded at: https://www.mdpi.com/article/10.3390/healthcare11101363/s1, Table S1: Stratified analysis by the severity of disability.

## Abbreviations

NHIRD: National Health Insurance Research Database; PST: Pap smear Test; PVS: Persistent vegetative state; CCI: Charlson Comorbidity Index; PSM: Propensity Score Matching; OR: Odds ratio; CI: confidence interval; NTD: New Taiwan dollars.
