# Supplementary material for: Comparison of Cervical Cancer Screening Used between Individuals with Disabilities and Individuals without Disabilities
Source: Healthcare (Basel). 2023 May 9;11(10):1363. doi: 10.3390/healthcare11101363 (PMC10217891; doi:10.3390/healthcare11101363)
Supplement: Supplementary file 1 [file healthcare-11-01363-s001.zip › healthcare-2346517-supplementary.pdf]

Supplement Table S1. Stratified analysis by the severity of disability

	Without disability vs. Mild				Without disability vs.				Without disability vs. Severe				Without disability vs. Profound			
	aOR	95% CI		p-value	aOR	95% CI		p-value	aOR	95% CI		p-value	aOR	95% CI		p-value
Disability type																
Without disability (ref)	-	-	-	-	-	-	-	-	-	-	-	-	-	-	-	-
Moving functional limitation	0.97	0.95	1.00	0.025	0.33	0.30	0.36	<0.001	0.65	0.62	0.67	<0.001				
Internal organ function loss and related	1.06	0.97	1.16	0.188	0.81	0.69	0.95	0.012	1.08	0.97	1.19	0.155	0.57	0.53	0.61	<0.001
Hearing impairment	1.00	0.94	1.05	0.863	0.86	0.79	0.95	0.002	0.95	0.88	1.02	0.157				
Multiple disabilities	0.90	0.61	1.33	0.599	0.43	0.39	0.47	<0.001	0.62	0.56	0.69	<0.001	0.50	0.47	0.55	<0.001
Chronic mental health conditions	1.12	1.06	1.19	<0.001	0.76	0.70	0.83	<0.001	0.81	0.78	0.84	<0.001	0.70	0.51	0.97	0.030
Visual impairment	0.87	0.80	0.95	0.002	0.69	0.62	0.76	<0.001	0.79	0.72	0.87	<0.001				
Intellectual and developmental disability	0.47	0.43	0.52	<0.001	0.30	0.26	0.33	<0.001	0.42	0.39	0.45	<0.001	0.19	0.15	0.22	<0.001
Dementia	0.63	0.48	0.82	0.001	0.26	0.17	0.42	<0.001	0.31	0.22	0.45	<0.001				
Vocal and speech impairment	0.86	0.74	1.00	0.057	0.56	0.42	0.74	<0.001	0.69	0.53	0.90	0.006				
Motion and balance impairment	0.73	0.52	1.02	0.068												
Facial disfigurements	1.23	1.00	1.52	0.050	1.05	0.58	1.90	0.866	1.05	0.71	1.56	0.816				
Intractable epilepsy	1.04	0.87	1.26	0.643												
Rare diseases	1.14	0.69	1.89	0.611												
Congenital disorders	0.53	0.28	0.99	0.049												
Others[a]	0.37	0.20	0.70	0.002	0.26	0.14	0.51	<0.001	0.41	0.25	0.65	<0.001	0.14	0.08	0.24	<0.001

*Model was controlled for five variables, including age, monthly salary, urbanization level of residence area, CCI, and dental calculus cleaning

a. Without disability vs. Mild disability: including autism, chromosomal abnormalities, and inborn errors of metabolism.

Without disability vs. Moderate disability: including autism, chromosomal abnormalities, inborn errors of metabolism, congenital disorders, motion, balance impairment, and rare diseases.

Without disability vs. Severe disability: including autism, chromosomal abnormalities, inborn errors of metabolism, congenital disorders, motion and

balance impairment, intractable epilepsy, and rare diseases.

Without disability vs. Profound disability: including visual impairment, vocal and speech impairment, moving functional limitation, dementia, autism, chromosomal abnormalities, inborn errors of metabolism, congenital disorders, and rare diseases.
